# Supplementary material for: Optimization and Molecular Mechanism of Novel α-Glucosidase Inhibitory Peptides Derived from Camellia Seed Cake through Enzymatic Hydrolysis
Source: Foods. 2023 Jan 13;12(2):393. doi: 10.3390/foods12020393 (PMC9857891; doi:10.3390/foods12020393)
Supplement: Supplementary file 1 [file foods-12-00393-s001.zip › foods-2115893-supplementary.pdf]

foods

Supplementary Materials

Optimization and Molecular Mechanism of Novel α-glucosidase Inhibitory Peptides Derived from Camellia Seed Cake through Enzymatic Hydrolysis

Table S1. Independent variables and code levels for the SCPH based on BBD.

Independent variables	Code units	Coded Levels		
		-1	0	1
Temperature (˚C)	A	45	50	55
pH	B	6.5	7	7.5
Protease concentration (U/g)	C	2000	4000	6000
Time (h)	D	3	4	5

Table S2. RSM design and results.

Std	Run	A:Temperature	B:pH	C:Time	D:Protease concentration	α-glucosidase inhibition activity/%
13	1	50	6.5	3	4000	48.16
4	2	55	7.5	4	4000	51.02
25	3	50	7	4	4000	58.9
18	4	55	7	3	4000	54.56
29	5	50	7	4	4000	59.33
1	6	45	6.5	4	4000	46.61
20	7	55	7	5	4000	53.91
27	8	50	7	4	4000	57.42
12	9	55	7	4	6000	47.96
9	10	45	7	4	2000	45.94
10	11	55	7	4	2000	45.46
8	12	50	7	5	6000	46.85
28	13	50	7	4	4000	57.39
15	14	50	6.5	5	4000	49.08
3	15	45	7.5	4	4000	51.32
26	16	50	7	4	4000	58.82
22	17	50	7.5	4	2000	38.61
2	18	55	6.5	4	4000	47.99
21	19	50	6.5	4	2000	35.57
23	20	50	6.5	4	6000	44.35
17	21	45	7	3	4000	54.65
16	22	50	7.5	5	4000	52.47
6	23	50	7	5	2000	40.23
19	24	45	7	5	4000	52.34
24	25	50	7.5	4	6000	48.1
11	26	45	7	4	6000	47.33
14	27	50	7.5	3	4000	51.82
7	28	50	7	3	6000	47.28
5	29	50	7	3	2000	42.08

Table S3. Details information of peptides with denovo scores > 90 used for virtual screening.

Peptide	Denovo Score	m/z	Mass	Docking score(Kcal/mol)
LLVLYYEY	94	538.287	1074.5637	-9.335
LLLLPSYSEF	92	591.3289	1180.6379	-9.06
LCDQCPPHA	91	549.229	1096.4429	-8.535
ATNPPCCQP	90	522.7232	1043.4165	-8.868
KDDFVEKR	99	518.7742	1035.5349	-8.307
TSCSSPSYPFQ	90	630.7604	1259.5129	-8.926
LVGDCQRY	95	505.7458	1009.4651	-8.216
LDMSAERGN	92	504.7228	1007.4342	-8.153
AEDLPRRC	93	508.7515	1015.4869	-7.913
LKEKLPGHT	97	511.8037	1021.592	-7.34
MKDAREQF	93	512.7495	1023.4807	-7.076
HRLDQCR	94	513.744	1025.4824	-6.68
VVRDPGSHY	95	515.2598	1028.5039	-6.652
SDGSGPHHGW	99	518.7155	1035.4158	-6.435
RVNEGETFS	94	519.7378	1037.4778	-6.325
LNHQSQNLDQK	96	662.8312	1323.6531	-6.325
EFNRSPYQ	91	520.7464	1039.4723	-5.93
KSSSAGPSQR	96	523.7649	1045.5151	-5.89
LPNYHPAPR	99	532.7872	1063.5562	-5.851
AEDLRPRGC	98	537.2609	1072.5083	-5.836
LLNDRETGR	94	537.2894	1072.5625	-5.834
LAGNPQRQQ	99	506.2695	1010.5257	-5.81
GLTQSQQCR	92	539.2593	1076.5032	-5.756
QQQPTTEPR	91	542.7732	1083.5308	-5.711
VVRSEAGDMK	96	546.2794	1090.5439	-5.653
KGPKSDPSYN	90	546.7681	1091.5247	-5.617
LYKDDFLDKR	92	656.8481	1311.6821	-5.611
LTDEHGHPVQ	99	566.7731	1131.5308	-5.605
PAGQDLPLLNA	94	575.8112	1149.603	-5.513
LLQGHDDRR	96	370.5295	1108.5737	-5.478
EDAALHMVPH	92	568.2648	1134.5127	-5.42
KETPAEGAPSK	97	557.7904	1113.5667	-5.277
TMEQQCRLQR	92	455.8883	1364.6289	-5.247
QAGAQPQHRL	94	553.2961	1104.5789	-5.245
LLSSSQPNTR	96	551.7962	1101.5779	-5.238
EHGHDTLPVQ	94	378.1844	1131.5308	-5.201
VTGKQPPGAEQ	97	556.2906	1110.5669	-5.156
LAGNPQRQAGQ	98	570.2983	1138.5842	-5.136
NEMTAGKLNR	95	575.2912	1148.5608	-5.089
LAGNQPHQQQ	95	560.777	1119.542	-5.006
SSDTLDNVKAK	95	589.3074	1176.5986	-4.927
VDEETAAANPR	95	586.7804	1171.5469	-4.922
EDYGNPPRKT	97	588.7856	1175.5571	-4.864
LRDPEREEQ	97	586.2878	1170.5627	-4.765

RLHQNLDER	95	394.2095	1179.6108	-4.729
TDELLSNDHNQ	93	643.285	1284.5581	-4.72
CSSPSYTRDQ	97	600.7509	1199.4878	-4.633
VSEETTTVGKR	91	603.8195	1205.6252	-4.622
TEESVTTGVKR	90	402.8821	1205.6252	-4.603
LFPEKGSPKSQ	93	609.3295	1216.645	-4.561
LKEKLPNHKD	99	611.3502	1220.6877	-4.345
LSPSTDCSKVH	98	615.793	1229.571	-3.679
